# Oral Microbes, Biofilms and Their Role in Periodontal and Peri-Implant Diseases

**DOI:** 10.3390/ma11101802

**Published:** 2018-09-22

**Authors:** Jérôme Frédéric Lasserre, Michel Christian Brecx, Selena Toma

**Affiliations:** Department of Periodontology, Université catholique de Louvain, 1348 Louvain-la-Neuve, Belgium; brecxparo@gmail.com (M.C.B.); selena.toma@uclouvain.be (S.T.)

**Keywords:** periodontitis, peri-implantitis, biofilms, oral bacteria

## Abstract

Despite many discoveries over the past 20 years regarding the etio-pathogenesis of periodontal and peri-implant diseases, as well as significant advances in our understanding of microbial biofilms, the incidence of these pathologies still continues to rise. This review presents a general overview of the main protagonists and phenomena involved in oral health and disease. A special emphasis on the role of certain keystone pathogens in periodontitis and peri-implantitis is underlined. Their capacity to bring a dysregulation of the homeostasis with their host and the microbial biofilm lifestyle are also discussed. Finally, the current treatment principles of periodontitis and peri-implantitis are presented and their limits exposed. This leads to realize that new strategies must be developed and studied to overcome the shortcomings of existing approaches.

## 1. Introduction

### 1.1. Microbes and Their Human Hosts

Humans are usually colonized from birth by many microbes that usually live in harmony with their host as commensal or symbiotic communities [1]. Among these, bacteria live in or on the human body, on mucosal surfaces or on the skin, and contribute in many ways to the host’s life [2]. Indeed, in the healthy state, the commensal microbiota plays a protective role, like an invisible shield, against exogenous pathogens. Microbes also participate in food digestion, contribute to the synthesis of certain vitamins, and can educate our immune system [2,3]. It is estimated that the number of bacteria covering the human body is ten times greater than that of the eukaryotic cells of which we are composed [2,4]. Humans have, most likely, co-evolved with these microbes that have provided us with genetic and metabolic attributes [5]. Consequently, they are defined as “metaorganisms” [3]. Most of the microorganisms in humans are located in the gastrointestinal tract, where their concentration reaches its highest level in the colon with approximately 10^11^–10^12^ cells/mL [6]. In fact, these indigenous microbes are usually essential in maintaining a healthy state, contrary to what was previously believed.

### 1.2. The Oral Microbiome

At the entrance of the upper digestive tract is the oral cavity (mouth), which is a very complex ecosystem that can harbor more than 150 different species of bacteria in one individual [7], as well as other types of microbes including archaea, fungi, protozoa and viruses [8]. More than 700 bacterial species have been isolated and identified from oral samples. They normally act as symbiotic communities with the host [9] but are also able to initiate a number of diseases in certain situations. The difference between a commensal and a symbiotic microbiota is subtle but important to clarify. The term commensal refers to partners that can live together but have no obvious mutual benefits [1]. A symbiosis is more than that; it is a relationship where both individuals (host and microbes) live in harmony and co-dependently. It is a real cooperation, a host–microbe mutualism [5]. For instance, periodontal pockets provide an ideal habitat for anaerobic proteolytic bacteria to grow, with anaerobic conditions and nutrients like peptides secreted in the gingival crevicular fluid [10]. Conversely, humans can also take advantage of the presence of oral microbes in various ways. First, commensals act as a natural barrier against exogenous or opportunistic pathogens. This barrier of resistance towards colonization is well illustrated when oral candidiasis develops after an antibiotic regimen [11]. Another example is the capability of the oral microbiota to metabolize inorganic nitrate from green vegetables, which is beneficial to the human body. Oral bacteria reduce inorganic nitrate into nitrite, which is then absorbed in the stomach before entering the blood stream [12]. There, it is transformed into nitric oxide, which is antihypertensive and vasoprotective [13]. Bacterial nitrite production by oral nitrate-reducing bacteria has also been shown to have antimicrobial effects against acidogenic bacteria such as *Streptococcus mutans* and to consequently reduce bacterial acid production and contribute to caries prevention [14]. These examples illustrate the mutual benefits between oral microbes and their human host.

Approximately 60% of the bacterial species that inhabit the oral cavity are not cultivable [15]. Culture-independent methods developed in the last two decades, such as checkerboard DNA–DNA hybridization or 16S rRNA gene sequencing, have provided considerable additional knowledge on the nature of the microbiotas associated with oral health and disease [16,17]. In the mouth, various types of tissues, growth conditions and nutrients are encountered in the development of different communities. Indeed, some bacteria are much more prevalent in some environments of the oral cavity than in others because they find ideal conditions to survive. For instance, the microbiota of the saliva resembles that of the tongue and differs significantly from that present on teeth and root surfaces [18,19]. Microbial community differences also occur between different people, even in health [15].

At present, the oral microbiota is one of the best-characterized microbiotas in humans because saliva and biofilms are easily harvested from oral surfaces. Its analysis is important for our understanding of its role in the development and pathogenesis of infectious oral diseases. It has been studied in various sites and conditions, such as around teeth or oral implants, and significant differences in its constitution have been demonstrated between health and disease states [15,17,20,21,22,23,24]. The principal findings of these studies showed that archaea, a group of single-celled microorganisms, were restricted to a small number of methanogen species, whereas more than 700 oral bacterial species belonging to different phyla (Actinobacteria, Bacteroidetes, Firmicutes, Proteobacteria, Spirochaetes, Synergistetes and Tenericutes and the uncultured divisions GN02, SR1 and TM7) were observed [8].

### 1.3. Oral Microbial-Shift Diseases

Oral health is linked to the equilibrium between the host and its commensal microbiota. Qualitative and/or quantitative shifts of the oral microbiome can lead to dysbiosis, an imbalance that is responsible for the development of microbe-related pathologies [25]. For instance, various oral diseases like periodontitis and peri-implantitis are strongly associated with dysbiotic microbial communities [26]. Some studies also reported a more relative but interesting association between some oral bacteria and *a priori* non-infectious disease like oral cancer. Hence, *Porphyromonas gingivalis* and *Fusobacterium nucleatum* have potential antigens like FimA and FadA adhesins that could lead to the development and progression of carcinomas (epithelial cell cancers) [27]. Additionally, a clinical study revealed an association between inadequate dental hygiene and an increased risk of oral cancer, especially in heavy alcohol consumers [28]. The authors of that report proposed that the risk could be related to the production of acetaldehyde; indeed, oral bacteria in saliva can metabolize ethanol into acetaldehyde, a known carcinogen. Furthermore, non-oral infections such as endocarditis, brain or lung abscesses, hip arthroplasty infections, and septicemias have also been correlated to oral bacteria that can access the blood stream through untreated caries lesions or via the periodontal/peri-implant pockets [29]. Finally, several systemic conditions like diabetes, preterm birth and cardiovascular diseases have been associated with periodontal disease and their microbiota in epidemiological studies [30,31,32]. Many human diseases are thus caused or influenced, directly or indirectly, by the oral microbiome. The present article will focus on two important infectious oral diseases: periodontal and peri-implant diseases.

## 2. Periodontal and Peri-Implant Diseases

### 2.1. Definition

Periodontitis and peri-implantitis are two major oral diseases that we have to deal with in periodontal practice. They are polymicrobial inflammatory diseases that lead to the destruction of the tissue supporting the tooth/implant. Without treatment, they result in tooth/implant loss.

### 2.2. Epidemiology

Periodontitis is one of the most frequent infections in humans and is often recognized as the leading cause of tooth loss in adults [25]. It can lead to oral and potentially systemic disabilities. Recent epidemiological data from the Global Burden of Disease (GBD) 2010 study suggest that periodontitis is the sixth-most prevalent condition in the world [33]. Its frequency has increased slightly since 1990 and ranges between 10.5% and 12% of the population, depending on the region [34]. Additional information from the National Health and Nutrition Survey (NHANES) 2009–2010 presents the periodontal health status of adults in the U.S.; nearly 4000 patients (aged >30 years) were examined, and periodontitis was observed in more than 47% of the sample [35]. More precisely, 8.7%, 30.0% and 8.5% had mild, moderate and severe periodontitis, respectively. The prevalence was significantly higher in older participants (periodontitis was present in around 25% of young adults versus 70% of patients older than 65 years).

The epidemiology of peri-implantitis, a biological complication of oral implants, is less well studied compared to that of periodontal diseases because of the relatively recent development of this disease. Two important Swedish cross-sectional studies of 662 and 216 subjects evaluated the prevalence of peri-implantitis on Brånemark System^®^ implants with a documented function time of at least 5 years. The recorded values of peri-implantitis were 28% and 16% for the studied patients, and 12% and 7% at the implant level, respectively [36,37]. However, it was stated at the sixth European Workshop on Periodontology, organized by the European Federation of Periodontology in 2008, that very few epidemiological data of peri-implant diseases were available and that research should be conducted in a way that establishes accurate estimations of the disease and associated risk factors [38]. Since then, several studies have evaluated the prevalence and incidence of peri-implantitis in various populations, implant systems and clinical situations [39,40,41,42,43,44,45]. The values varied widely between the studies, from 9% to 47% at the patient level. These differences were partly due to the definition of peri-implantitis, which differed between the studies, and to the mean function time of the implants. The incidence of peri-implantitis tended to increase in patients without supportive therapy [46] and with time of function [42]. A recent systematic review, which evaluated the current epidemiology of peri-implant diseases, retained only 15 articles reporting on the topic and meeting the inclusion criteria [47]. In that review, no limits on function time were applied but at least 100 patients had to be included and subject-level data had to be reported for the study to be eligible. Weighted mean prevalence of mucositis and peri-implantitis at the patient level was 43% and 22%, respectively. Although the first consequence of these biological complications is implant loss, the systemic effects of such infections are still unknown.

### 2.3. Etiology

**Figure 1:**
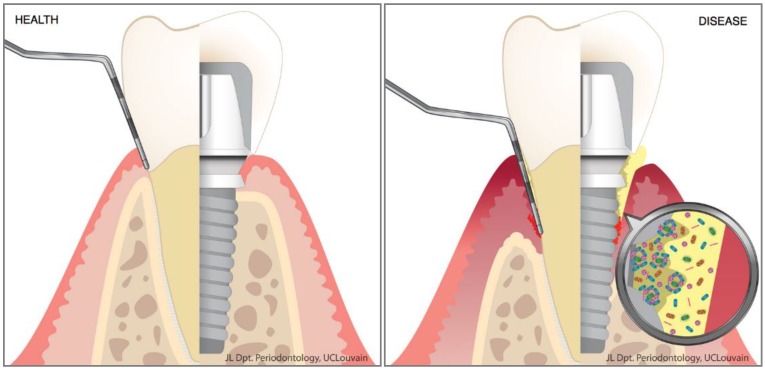
Periodontal/peri-implant tissues in health and disease. In the diseased state, the dysbiotic oral biofilm (yellow) that accumulates on the tooth/implant surface is responsible for the destruction of the supporting tissues through unresolved inflammation. This leads to the formation of periodontal/peri-implant pockets.

An emerging concept is the strong association between oral dysbiosis and oral disease [48]. In the healthy mouth, teeth are surrounded by the periodontium. This entity represents the tooth-supporting tissues and is composed of five elements: the gingiva, the alveolar mucosa, the alveolar bone, the periodontal ligament and the cementum (Figure 1). Each of these components is essential for maintaining the proper attachment and function of the teeth. All the structures found in the mouth (including teeth and implants) are permanently soaked in saliva containing billions of microorganisms (bacteria, viruses, archaea, protozoa, fungi; 10^8^ cells/mL) [49]. In the healthy mouth, conditions are appropriate for these microbes, which live in harmony with the host and participate in many physiological reactions. Using various saliva proteins, they can adhere to biotic and abiotic surfaces, and form oral biofilms. On mucosal surfaces, the shedding mechanism occurring during oral epithelial turnover is a natural effective means of reducing microbial adhesion. But this protective phenomenon does not occur on tooth or implant surfaces, where the dental biofilm can accumulate in the periodontal/peri-implant crevice and stay in contact with the gingival epithelium (Figure 1).

In susceptible patients, if dysbiotic, these sticky microbial communities elicit an inflammatory host response that can damage the surrounding tissues including the alveolar bone. The precise pathogenic pathways that lead to tissue destruction are still poorly understood but research conducted during the past decade has provided significant insight into this old enigma [25,50]. For example, the dysbiotic oral microbiota involved in these pathologies can induce direct tissue destruction through proteolytic enzymes. Additionally, the periodontal/peri-implant tissues will be damaged because of a non-resolving innate and acquired immunity response [25].

The microbial etiology of periodontal disease was first proposed in the late 1800s, when the germ theory changed the world’s understanding of disease. However, specific pathogens remained elusive at this time, which led in the mid-1920s–1930s to the suggestion of other causes like trauma or disuse atrophy. Then, in the late 1950s, when it was observed that gingival inflammation resolved after routine cleaning and dental plaque removal, the belief returned that microbes were non-specifically involved in the etiology of periodontal disease: the so-called “non-specific plaque hypothesis” [51]. This theory placed importance on the entire community as a causative entity, rather than potential specific periodontal pathogens. Later, in the 1970s and 1980s, detailed cultural studies characterized dental plaque bacterial composition and revealed significant differences between healthy mouths and those with periodontitis [52]. This led to the “specific plaque hypothesis”, in which the disease was strongly believed to be associated with the presence of certain pathogenic microorganisms [53] because these species were not (or were hardly) detectable by culture in healthy subjects. Considerable research efforts were then engaged to identify pathogens responsible for periodontal disease, and molecular technologies allowed the manufacturing of DNA probes. In 1998, a landmark study was performed in 185 volunteers (25 healthy; 160 with periodontitis) using whole genomic probes and the DNA–DNA checkerboard hybridization technique [16]. The researchers collected 13,261 subgingival plaque samples and identified three major pathogenic bacteria that were very often encountered together and strongly associated with severe periodontitis. These bacteria, namely *Porphyromonas gingivalis*, *Tannerella forsythia* and *Treponema denticola*, were called the “red complex” bacteria and accepted as strong etiological agents of periodontal disease. However, although these periopathogens were identified as potential causative agents of periodontitis at this time using microarray techniques, data collected since the early 2000s, during a period that saw enormous advances in microbiome characterization—first with Sanger sequencing and then with next generation sequencing—demonstrated that the situation is much more complex than that. Indeed, many works during the past 15 years have focused on the precise characterization of microbial profiles associated with oral health, and periodontal and peri-implant diseases using these novel technologies that sequence the bacterial 16s rRNA gene for microbial identification [15,17,21,23,24,54]. New information came out of these studies: first, the well-known microorganisms of the red complex could be found in sites and subjects in the absence of disease; second, new potential periopathogens emerged, some of which were not necessarily Gram-negative (*Filifactor alocis*, *Peptostreptococcus spp.*). These new candidates also outnumber the classical red complex species in the diseased sites but their pathogenic properties remain to be discovered [55].

The current model of periodontal/peri-implant disease, the “polymicrobial synergy and dysbiosis” model, tries to integrate the numerous theories from the past. It is based on the hypothesis that disease is provoked by a dysbiotic community shaped progressively by the introduction (even at low abundance) of keystone pathogens like *Porphyromonas gingivalis* [56]. In some clinical situations (physical disruption of the epithelium, antibiotic regimen, pathogen infection, host genetic defects, bacterial gene modification, tobacco smoking), these kinds of pathogens could colonize and develop into the commensal community by immune subversion, and then influence the whole symbiotic microbiota to become more pathogenic and initiate disease [20,48]. The microbiota is then progressively shaped by environmental changes into a more inflammophilic community composed of large proportions of pathobionts capable of maintaining dysbiosis and subsequent disease [57,58,59]. This model combines the previous “polymicrobial disruption of homeostasis” [25] and the “keystone pathogen hypothesis” [60]. According to this model, the key pathogens do not directly cause disease (as specific pathogens), but manipulate, through bacterial communication, the commensal microbiota that globally changes its metabolic activities to increase its pathogenicity.

### 2.4. Microbial Ecology of Dental Plaque

**Figure 2:**
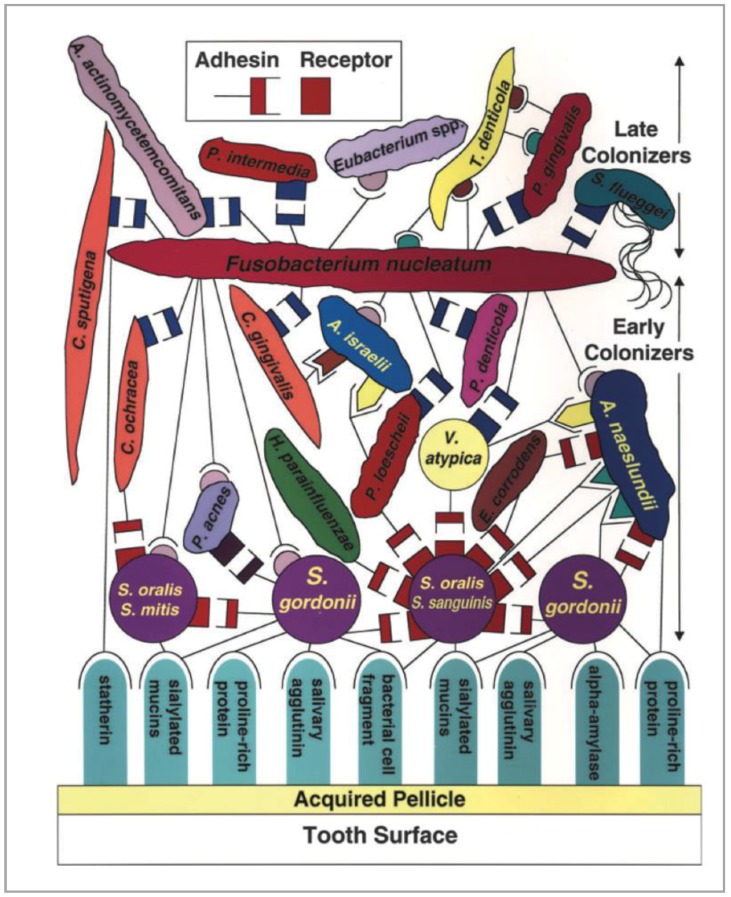
Intergeneric coaggregation among oral bacteria [64].

Saliva contains thousands of free-floating bacteria per milliliter that progressively deposit and adhere to dental/implant surfaces, first by non-specific physicochemical means and then by specific interactions with surface-adsorbed saliva proteins. The initial colonizers of early dental plaque in the first few days are essentially composed of Gram-positive bacteria, mostly cocci. The population then becomes increasingly complex, shifting progressively to a largely Gram-negative community with the appearance of rods, filamentous organisms, vibrios and spirochetes [61]. This maturation of undisturbed dental plaque is very important because it is associated with the clinical development of gingival and peri-implant mucosal inflammation [62,63]. This microbial succession is mediated by coaggregation between different bacterial species that corresponds to intergeneric specific cell-to-cell recognition via surface adhesins and receptors (Figure 2) [64].

Later, in more advanced disease states such as periodontitis, the diversity of the periodontal microbiota increases further. It is composed supragingivally of a dense filament-containing plaque and subgingivally of flagellated bacteria, spirochetes and small Gram-negative bacteria [65]. More recent studies analyzing the initial composition of early dental plaque have confirmed, with molecular techniques, that most of the early colonizers were Gram-positive and belonged to the genera *Streptococcus spp.* and *Actinomyces*. Some Gram-negative genera, like *Neisseria* (aerobes) or *Veillonella* (anaerobes), were also observed [66,67]. Nevertheless, these culture-independent methods have demonstrated that even in healthy situations and in early dental plaque, some periopathogens like those of the red complex or *Aggregatibacter actinomycetemcomitans* could be found. This was also the case in the pockets of newly abutment-connected dental implants [68]. Surprisingly, these studies highlighted real differences in the microbial profiles of the participants, demonstrating a significant subject-specificity of the initial dental plaque biofilm.

**Figure 3:**
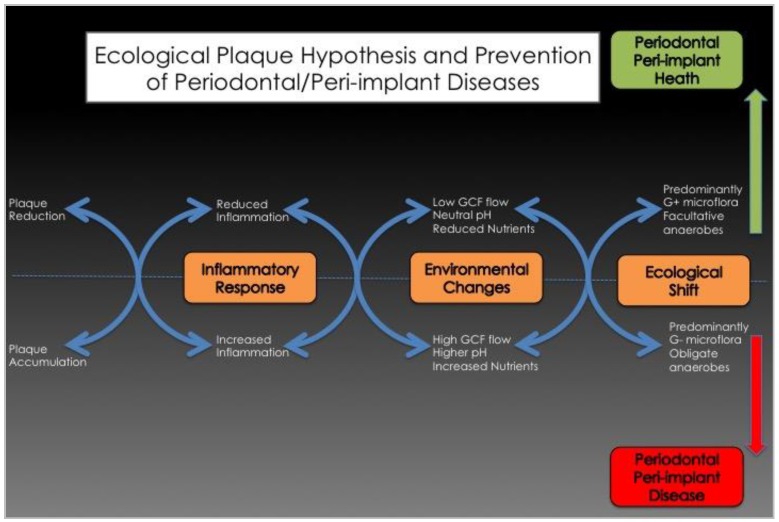
The ecological plaque hypothesis (Adapted from [69]).

After a few days, the accumulation of dental plaque biofilms in the periodontal or peri-implant sulcus induces clinical signs of inflammation, including increases in probing pocket depths, gingival index and gingival crevicular fluid (GCF) flow [62,63]. Thus, the early colonizers, mainly Gram-positive aerobes composed of *Streptococcus spp.* and *Actinomyces spp.*, influence the local environment, which, in turn, becomes suitable for secondary colonizers such as *Fusobacterium nucleatum*. This bacterium acts as a “bridging species”. Indeed, through coaggregation, it allows the adhesion of late colonizers and periopathogens like *Porphyromonas gingivalis* [10,64]. This succession during the colonization of the periodontal/peri-implant crevice shows how the accumulation of commensal bacteria can induce (if important and undisturbed) a change in the local habitat (↑pH, ↑GCF, ↓Eh (Redox potential), ↓O_2_) that allows periopathogens to colonize the periodontal/peri-implant crevice. This shift from a symbiotic microbial community to a more complex and aggressive microbiota is a risk predisposing the site to disease. This sequence is in accordance with what has been called the “ecological plaque hypothesis” of periodontal disease (Figure 3) [69].

It is now well accepted that dental biofilms play a key role in the initiation and progression of periodontal and peri-implant diseases. However, the precise mechanisms leading to homeostasis disruption and the detailed pathways of pathogenesis are still unclear.

### 2.5. Pathogenesis of Periodontal and Peri-Implant Diseases

**Figure 4:**
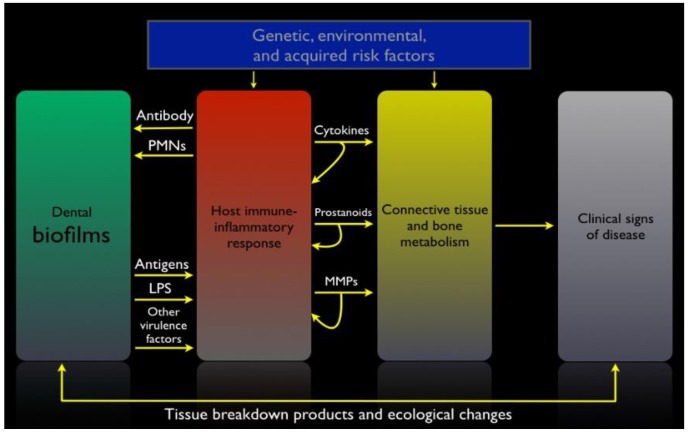
Schematic of the pathogenesis of periodontal and peri-implant diseases (PMNs: polymorphonuclear neutrophils; MMPs: matrix metalloproteinases; LPS: lipopolysaccharides). (Adapted from [75]).

Although the etiology of periodontal and peri-implant diseases is bacterial, and some well-characterized pathogens display destructive virulence factors, the pathogenesis of periodontitis and peri-implantitis is essentially mediated by the host response [70]. Certain advances in the past 15 years instilled a new appreciation of pathogenesis. Indeed, it has been demonstrated that even in health, the periodontal or peri-implant tissues that are in close contact with the dental biofilm show an active immune response, which is physiological. This low-grade inflammation is complex and involves both innate and acquired immunity as well as the complement system, the major link between the two arms of the immune system [71,72]. Dysregulation in the production of inflammatory mediators in response to the dysbiotic microbial challenge leads to the production of toxic products by the host cells. When produced in excess, these toxic products are responsible for tissue destruction around teeth and oral implants [70]. Additionally, the identification of Toll-like receptors (TLRs) highlighted how both commensal and pathogenic bacteria can initiate innate immune responses [73,74]. Finally, the discovery that most bacteria live in biofilms as tenacious multicellular communities has been important for our understanding of how microorganisms could resist the host immune response and even some conventional anti-infective approaches [25]. Figure 4 illustrates the pathogenesis of periodontal and peri-implant diseases.

**Figure 5:**
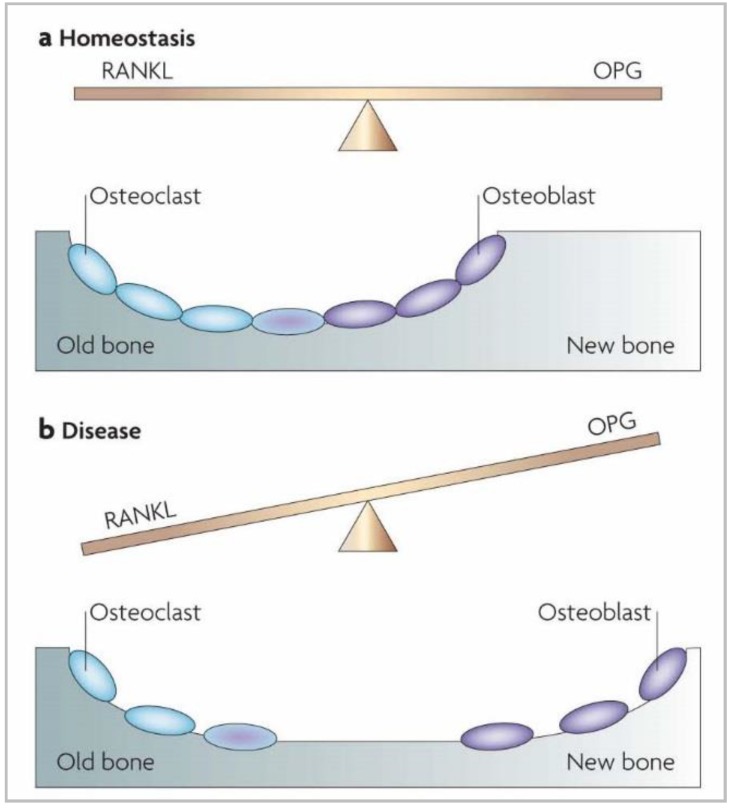
The NF-κB model of bone resorption during periodontal and peri-implant diseases [25].

To cope with constant contact with microorganisms and their virulence factors in the periodontal and peri-implant pockets, the host orchestrates the expression of defense mediators. First, direct recognition of bacteria (or virulence factors) by resident cells occurs through interaction between the TLR and bacteria, and this mediates the production of chemokines. Thereafter, intercellular adhesion molecules and E-selectin are produced at the surface of local endothelial cells. These molecules initiate the transit of polymorphonuclear neutrophils (PMNs) from the gingival vessels to the junctional epithelium, where they act as the first line of defense in the periodontium and peri-implant mucosae. This migration is guided by a gradient of interleukin (IL)-8, a cytokine produced in abundance by gingival epithelial cells [76]. PMNs are essential in maintaining periodontal health, as individuals with congenital diseases characterized by a deficiency of PMNs, such as leukocyte adhesion deficiencies or neutropenia, systematically develop periodontal diseases [77]. To eliminate aggressive pathobionts, PMNs employ various antimicrobial strategies, including phagocytosis, reactive oxygen species production and intra- or extracellular degranulation of specific enzymes [78]. In addition to IL-8, the host also expresses other mediators that contribute to innate immunity and that are encountered in the gingival or junctional epithelia. Of these innate molecules, β-defensins, CD14 and lipopolysaccharide-binding protein play a role in the neutralization of oral pathogens as well as TLRs [74] and neutrophil extracellular traps (NETs) [79]. TLRs are host-cell receptors that recognize commensal and pathogenic microbes and launch immune reaction pathways to defend the host against microbial invasion. NETs form a web-like structure of decondensed nuclear chromatin or mitochondrial DNA that is released in the extracellular spaces by PMNs and is associated with an array of antimicrobial molecules, including peptides. Their aim is to eliminate invading periodontal or peri-implant pathogens. In parallel to this innate immunity, periodontal and peri-implant tissues produce numerous cytokine and chemokine molecules that, in a refined equilibrium, help maintain a healthy situation. However, some of them—like IL-1β, tumor necrosis factor (TNF)-α, IL-6 and IL-17—are known as strong pro-inflammatory molecules that, without appropriate control, can lead to tissue destruction. Indeed, these signaling molecules stimulate the activation of enzymes and transcription factors that in turn recruit more immune cells and degrade the surrounding tissues by maintaining a continual loop of local inflammation [71,75]. Three protein pathways—nuclear factor kappa B (NF-κB), cyclo-oxygenase (COX) and lipo-oxygenase (LOX)—are activated in periodontal and peri-implant diseases and play key roles in maintaining inflammation and bone resorption. COX and LOX produce lipid mediators such as prostaglandins and leukotrienes (eicosanoids) by the oxidation of arachidonic acid. These lipid signaling molecules are pro-inflammatory, and the consequence of the prolonged elevation of their concentration is alveolar bone resorption [70]. The NF-κB pathway is another system probably related to bone resorption in periodontitis and peri-implantitis. Normally, the mechanisms that regulate bone deposition and resorption during remodeling are mediated through the refined equilibrium between the expression of two molecules: receptor activator of NF-κB ligand (RANKL) and osteoprotegerin (OPG) [25]. Indeed, RANKL, produced by several cell types, interacts with its receptor, RANK, located on the membrane of osteoclast precursors. This interaction allows them to finish their differentiation into active osteoclasts that will resorb the alveolar bone. OPG is a soluble RANKL receptor that is secreted by osteoblasts and, at high concentrations, prevents RANK–RANKL interaction and limits bone resorption. OPG formation is regulated by transforming growth factor β, and RANKL is induced by pro-inflammatory cytokines like IL-1β and TNF-α. Together, this demonstrates how the production of pro-inflammatory mediators can influence the RANKL/OPG ratio and contribute to periodontitis and peri-implantitis (Figure 5).

Periodontitis and peri-implantitis also involve the destruction of the connective tissues including collagens, proteoglycans and other components of the extracellular matrix. The degradation of this extracellular matrix is performed by matrix metalloproteinases, a group of enzymes including collagenases. They are released locally by immune cells (macrophages or PMNs) or resident tissue cells (mostly gingival fibroblasts, because of their high number) [75]. All these immune and inflammatory reactions that lead to periodontal and peri-implant diseases are induced by the microorganisms that develop on enamel and titanium surfaces as microbial biofilms in close contact with the junctional and sulcular epithelia of their host. The biofilm mode of growth of dental plaque is likely to influence the pathogenicity of oral microbes.

## 3. Current Treatment Principles of Periodontitis and Peri-Implantitis

Despite all the discoveries of the past 20 years regarding the etio-pathogenesis of periodontal and peri-implant diseases, as well as the significant advances in our understanding of microbial biofilms, the incidence of these pathologies continues to rise [34,80]. Even though peri-implant diseases present some physio-pathological specificities [81,82] histo-pathological particularities [83] and are associated with microbiomes that seems to differ from those of periodontitis [23,84], they are recognized as biofilm-induced inflammatory diseases. They are often, and in many ways, compared to periodontitis. Both diseases are thus related to a switch from a symbiotic to a dysbiotic microbiota [56].

The main objective of peri-implantitis treatment is hence anti-infective as it is for periodontitis [85]. Control of the subgingival dysbiotic dental biofilm to restore homeostasis between the microbial community and its host remains the main purpose of currently available clinical treatments for these pathologies. This primarily involves giving instructions for proper oral hygiene, as well as nonsurgical mechanical debridement of the periodontal and peri-implant pockets. If performed carefully, these noninvasive mechanical therapeutic approaches most often allow the control of inflammation and disease in periodontitis [86]. Unfortunately, for advanced lesions with probing pocket depths of ≥7 mm, these treatments are less efficient, with about 15% showing no improvement [87]. Results of such treatment on furcation-involved teeth are also less beneficial, requiring more aggressive (surgical flaps) or alternative anti-infective approaches [88].

Few studies have investigated the efficacy of treatments of peri-implantitis lesions, but recent clinical trials and systematic reviews have shown that a nonsurgical mechanical approach is not sufficient for controlling the disease [89,90,91,92,93]. The relative failure of mechanical treatments in these clinical situations can be related to the fact that the disinfection is insufficient, leading to recolonization of the affected pocket and continued disease progression. Some local factors, such as a deep pocket, unfavorable root anatomy, or a rough surface threads, can explain the difficulty of achieving complete and efficient mechanical debridement. The recolonization of the periodontal or peri-implant pocket by a dysbiotic community can also come from pathogens that had previously infiltrated the dentin tubuli [94] or the periodontal/peri-implant tissues [95]. To improve clinical results, some authors proposed the use of conventional antibiotics or local antiseptics as adjunctive methods to the mechanical debridement of diseased pockets. Indeed, the clinical benefits of antibiotics used in this way have been demonstrated, and their use is now recommended for the treatment of aggressive periodontitis [96]. The use of antibiotics has also been suggested for severe periodontitis, though only when necessary, as the body of evidence for this is weaker [97]. However, considering the increase in incidence of microbial resistance (and the mild risk of the patient developing an allergy), the use of antibiotics should be kept to a minimum.

Subgingival irrigation as a nonsurgical treatment of periodontal and peri-implant diseases remains controversial [98], with a lack of randomized controlled clinical trials, and data that do not allow the discrimination between the relative efficacy of various available methods [99]. Therefore, the development of new strategies to better treat severe periodontitis and peri-implantitis is still needed. To this end, two distinct approaches can be considered. The first would try to improve the host’s immune/inflammatory response to the microbial challenge. The second would investigate new pre-clinical and clinical strategies to control more efficiently the periodontal and peri-implant biofilms and pathogens associated with periodontitis and peri-implantitis.

## 4. New or Recent Antibiofilm Strategies

**Figure 6:**
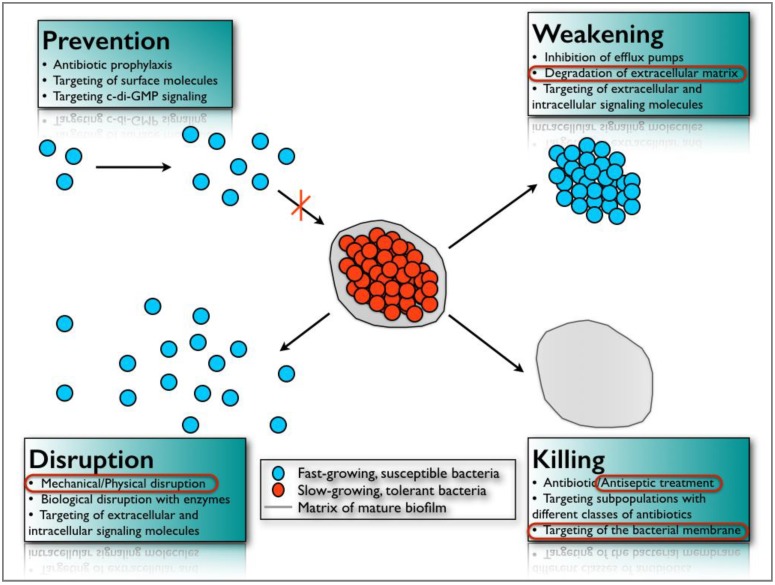
The four anti-biofilm strategies. (Adapted from [102]).

It is estimated that 99% of bacteria on earth live in biofilm aggregates [100] and most infectious diseases (65%) are related to the development of such sessile communities [101]. To counteract the natural resistance/tolerance of microbial biofilms against antimicrobial agents and to mitigate their pathologic consequences, new strategies are being considered and studied. They can be classified into four main categories according to Bjarnsholt et al. [102]: (a) prevention, (b) weakening, (c) disruption, and (d) killing (Figure 6).

The first approach, (a), aims to prevent biofilm formation through antibiotic prophylaxis or by modifying surface characteristics using antimicrobial or anti-adhesive coatings. For instance, silver nanoparticles on titanium surfaces are currently being evaluated for use in dentistry [103]. 

Weakening, (b), refers to interfering with signaling molecules, virulence factors and/or biofilm-forming properties to make the biofilm more susceptible to conventional antimicrobial agents and to the natural host defense system. To achieve this, quorum sensing inhibitors, inhibitors of small RNAs (messengers involved in biofilm formation), specific antibodies against virulence factors, or metabolically inactive metal ions that can interfere with iron metabolism are being tested in vitro [102]. 

The third antibiofilm approach, (c), aims to disorganize the biofilm structure in order to disrupt the communication network between its cells and to make them more susceptible to antimicrobials. For example, Alhede et al. [104] showed that vortexing *Pseudomonas aeruginosa* biofilms was a valuable in vitro method of increasing their sensitivity towards tobramycin. Another method for the same purpose is to use biological molecules such as enzymes (DNAses or dispersin B) that target the extracellular matrix components of the biofilm that are responsible, in part, for its cohesive property [105,106]. Nevertheless, the most efficient method for treating a biofilm infection is by mechanically or surgically removing the biofilm. Two basic examples of biofilm removal are tooth brushing and scaling, and root planning. These mechanical methods are the first choice when the contaminated surface can be accessed. Unfortunately, this is not always possible and innovative antibiofilm approaches need to be developed. 

The fourth strategy for controlling microbial aggregates, (d), is by killing biofilm cells by specific and/or nonspecific anti-infective means. New synergistic approaches involving small molecules (2-aminoimidazoles) capable of dispersing biofilm bacteria, and conventional antibiotics, have been proposed to better combat biofilm aggregates [107]. Furthermore, the use of bacteriophages (viruses that contaminate bacterial cells) [108], antimicrobial peptides [109] or photodynamic therapy [110] have also been investigated in the past decade as innovative antibiofilm strategies. Finally, among the numerous antibiofilm approaches that have been suggested, an interesting electrical enhancement of the effects of several antibiotics and industrial biocides have been described against different types of bacterial biofilms [111,112,113]. Electric currents have also been reported to have a bactericidal effect [114] in addition to detaching adherent bacteria [115] and preventing their adhesion [116]. The mechanisms by which these phenomena occur have not been elucidated in detail, but may be related to a better diffusion of antimicrobials through the biofilm [117], the electrolytic generation of oxygen [118] or the electrochemical formation of antimicrobial oxidants [119]. Electric currents might also lead to the degradation of the bacterial membrane, resulting in the leakage of the cytoplasmic constituents [114]. They could potentially have another physical effect on the charged extracellular matrix, which would induce a weakening of its structure. These antibiofilm phenomena, which have been described as bioelectric and electricidal effects, could be of great interest to the field of oral care, particularly in the treatment of advanced or refractory periodontal and peri-implant infections.

## 5. Concluding Remarks

Periodontitis and peri-implantitis are two major biofilm-induced inflammatory diseases that lead to the loss of teeth and oral implants if left untreated. These diseases are also associated with systemic conditions such as diabetes or cardiovascular diseases. Unfortunately, currently available treatments are not always successful, so innovative antibiofilm approaches need to be developed. Many strategies have been developed in that way and tested in vitro, as outlined above, but their clinical applicability is sometimes difficult. Considerable research efforts are needed, and additional anti-inflammatory approaches should be investigated to improve treatment efficacy.
